# Effects of Kinesio taping on skin deformation during knee flexion and extension: a preliminary study

**DOI:** 10.1186/s12891-022-05148-5

**Published:** 2022-02-28

**Authors:** Fei He, Xiaoxuan Wang, Menglian Yu, Yiyi Chen, Bo Yu, Jianqiang Lu

**Affiliations:** 1 Key Laboratory of Exercise and Health Science of Ministry of Education, School of Kinesiology, Shanghai University of Sport, No. 200 Hengren Road, Yangpu District 200438 Shanghai, China; 2 Department of Rehabilitation Medicine, Shanghai General Hospital, Shanghai Jiao Tong University School of Medicine, Shanghai, China; 3 Department of Rehabilitation Medicine, Shanghai Jiao Tong University Affiliated Sixth People’s Hospital, Shanghai, China

**Keywords:** Kinesio taping, Skin deformation, Motion capture systems, Skin biomechanics

## Abstract

**Background:**

Kinesio Taping (KT) is proved useful to many musculoskeletal disorders. But the mechanism remains unclear. The kinesio tape works by sticking to the skin surface. So exploring the interaction between the tape and the skin and analyzing its biomechanical influence may be an effective way to explore the mechanism of the tape.

**Objectives:**

This study aimed to investigate the effect of Kinesio taping and taping methods on skin deformation during knee joint flexion and extension motion and further explore its possible functional mechanisms.

**Methods:**

Ten healthy and pain-free subjects (4 males, 6 females) were recruited in this study. The skin observation area on the anterior side of the right thigh of the subjects was divided into 11 segments by 12 reflective marker points for distance measurement, from the distal knee to the proximal knee, the length of the interval was L1 to L11, and the total length was L0. Subjects were treated with no KT (NT), resting positive taping (RPT), resting negative taping (RNT), stretching positive taping (SPT), and stretching negative taping (SNT). A Qualisys infrared high-speed three-dimensional spatial coordinate capture system was used to observe changes in the length of the observed skin surface on the right anterior thigh during right knee flexion and extension in the sitting position.

**Results:**

During right knee flexion and extension in the seated position in 10 subjects, all skin segment deformations produced significant differences between intervention groups (*P* < 0.05), except for L1 during flexion (*P* = 0.07). During right knee flexion and extension, total length, L0, and spacing lengths, L1, L6, and L11, were longer in the NT group than in all other groups. L0 and L1 were both longer in the stretched position than in the rest position; L11 also showed this trend.

**Conclusions:**

The usage of the KT had an effect on the biomechanical changes of the skin, resulting in changes in skin deformation. I-tape, natural tension taping can shorten the skin distance between the two ends of the tape. Limb position during taping may influence the KT’s effects. However, the change in taping direction showed no significant effects on skin deformation during exercise. KT may apply a pre-stress in the biomechanics of the skin.

## Background

The skin is the largest organ of the human body and plays an important role in body functions, such as sensation, thermoregulation, and organism protection [1, 2]. Many physiological functions of the skin depend on its biomechanical properties, such as viscoelasticity, tension, and pressure resistance [1, 2]. The development of skin viscoelasticity measurement techniques has evolved from *ex vivo* to *in vivo* and from static to dynamic loading [3–5]. Many low-invasive or non-destructive *in vivo* testing methods have been widely used, including small-scale motion analysis techniques such as motion capture systems, which are technical devices used to accurately measure the motion of a moving object in three dimensions [6]. It records the motion of a moving object in the form of an image through several video capture devices arranged in space and then uses a computer to process the image data to obtain the spatial coordinates (X, Y, Z) of the different objects in different units of time measurement. Small three-dimensional motion capture systems combined with inverse finite element analysis methods have been successfully used for the *in vivo* measurement of small-scale deformation of human skin [7].

Kinesio taping is a non-invasive treatment technique in which an elastic patch is applied to the skin in a specific way to produce biomechanical and neurophysiological effects that are conducive to musculoskeletal system function and motion [8]. Kinesio tape was first used in the sports field since the 1970s. Traditional theories believe that it has various functions such as reducing pain, relaxing muscles, guiding fascia, and increasing proprioception [9, 10]. In recent years, studies have shown that [11–13] as a simple and effective adjuvant therapy for musculoskeletal diseases, kinesio taping has the effect of reducing pain and is a convenient choice for low back pain intervention. But there is also a meta-analyses showing [14] short-term kineso taping in reducing pain and increasing function in myofascial pain syndrome, shoulder impingement syndrome, patellofemoral pain syndrome is not significantly better than conventional physical therapy.

There are various approaches to Kinesio taping such as “muscle application,” “fascial application,” and “space tape,” among others [15]. Despite the widespread application of this technology, evidence regarding its functional mechanisms remains inconsistent [16]. In fact, tape is applied to the skin surface. The skin is a viscoelastic organ and the tape itself is viscoelastic; therefore, exploring the mechanical relationship between the tape and skin may suggest how to study the functional mechanisms of Kinesio taping. Dr. Kase, the founder of Kinesio taping, hypothetically explained the functional mechanisms by which the mechanical force applied to the local skin by the tape can cause the skin to crease, lifting the local skin to increase subcutaneous tissue gaps, which promotes local blood and lymphatic circulation and reduces the levels of pain-causing substances [17]. Other scholars have reported that Kinesio taping can effectively increase the subcutaneous tissue space after autopsy and ultrasound observation [18–20]. Most of these studies focus on the changes of subcutaneous tissue space, local skin temperature or blood flow, etc., and there are few direct studies on the effect of taping on skin deformation, and there is no observational study of the effect of taping on skin deformation during exercise. Based on the previous literature, we hypothesized that taping to lift the local skin may limit the skin deformation during its movement, and different taping methods may also affect the degree of this restriction.

Small three-dimensional capture systems are an advanced attempt to measure the full-field deformation of a local area of human skin *in vivo*. Therefore, with the development of optical measurement systems in the field of motion, it is possible that conventional motion capture systems can be used to observe skin deformation and the interaction between Kinesio taping and the skin. In this study, we observed skin deformation during exercise, compared the changes with or without Kinesio tape applied using different approaches, and further analyzed the effects of Kinesio taping on skin biomechanics. Moreover, we explored the possible mechanisms of Kinesio taping and value of conventional motion capture systems in skin biomechanics research. Our studies may provide insights into the mechanism by which Kinesio taping affects the biomechanics of the skin.

## Methods

### Subject recruitment

Healthy adult volunteers who met following criteria were included in this study: (1) aged 20–40 years; (2) physically healthy; (3) able to move normally; (4) no sports-related injuries; (5) no hip pain, knee pain, or other related discomfort; (6) no local skin infections or other contraindications to Kinesio taping; and (7) no cognitive impairment. The exclusion criteria included the following: (1) patients with cardiovascular, neurological, or psychiatric disease or severe visual or hearing impairment; and (2) patients with orthopedic disease of the spine (e.g., spinal sac herniation) or extremities (e.g., fractures, prostheses, osteoarthritis).

### Equipment and reflective marker setting

**Fig. 1:**
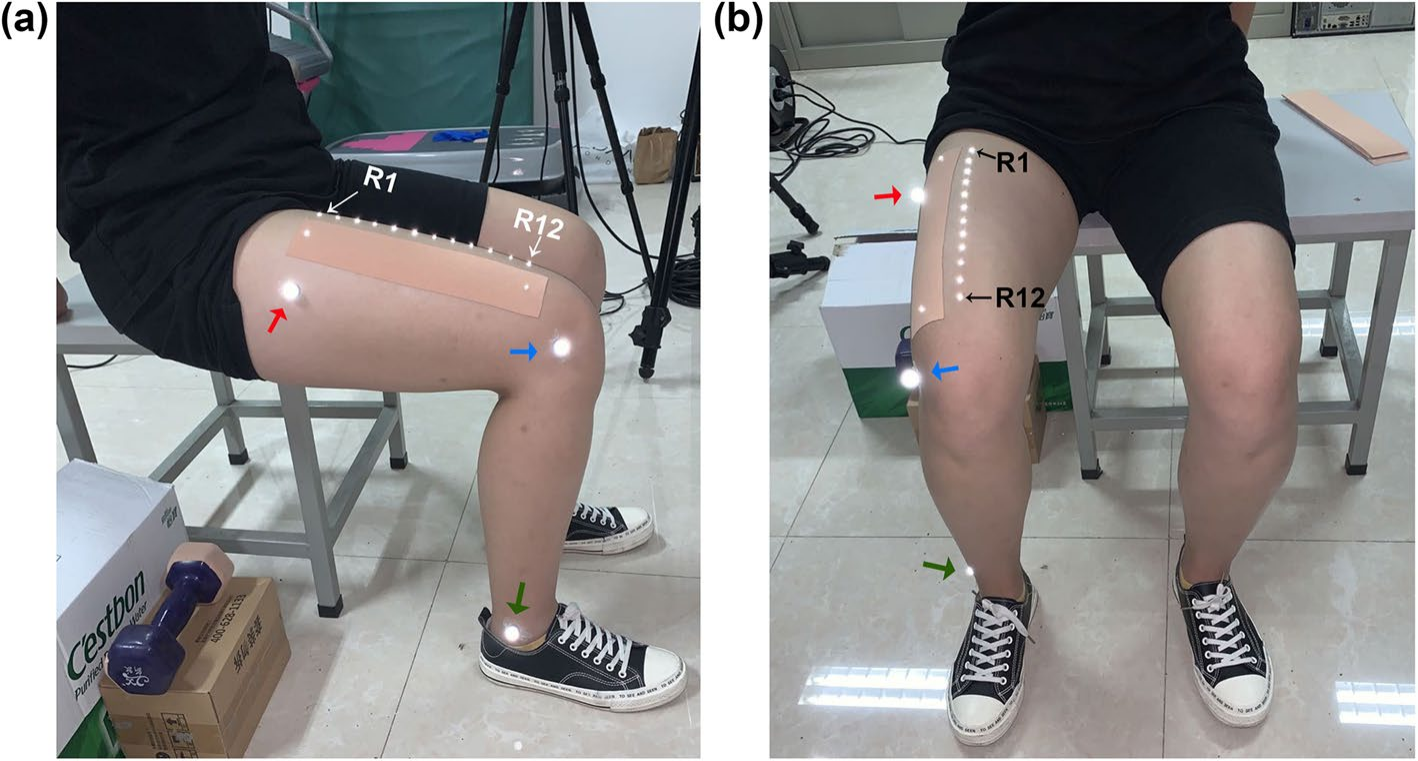
Reflective markers and the subject’s posture. (**a**) Medial view. (**b**) Frontal view. Reflective marker (diameter: 2.5 mm) positions (R1–R12; distance between two adjacent points: 20 mm); reflective markers (diameter: 10 mm) on the outer ankle (green arrows), condyles lateralis tibiae (blue arrows), and upper 2/3 of the line between the lateral condyle and greater trochanter (red arrows)

The deformation of the skin on the right anterior thigh during movement was measured in 10 subjects using a three-dimensional Qualisys infrared high-speed three-dimensional spatial coordinate capture system with a sampling frequency of 1000 Hz (Qualisys AB, Göteborg, Sweden). A total of 12 reflective markers (diameter: 2.5 mm) were affixed to the skin at 20-mm intervals along the line between the midpoint of the right groin and midpoint of the right femoral condyle (Fig. 1a-b). The 12 points were marked as R1–R12. L0 was used to refer to the total distance between R1 and R12. The distance between two adjacent marker points was referred to as L1 to L11. The reflective markers (diameter: 10 mm) were placed on the skin of the ipsilateral lateral ankle, lateral tibial condyle, and upper 2/3 of the line between the lateral condyle and greater trochanter to mark the angle of movement (Fig. 1a-b). Kinematic data were digitized using QTM software (Qualisys).

### Procedures

The subjects were seated on a stool to perform right knee flexion and extension exercises, that is, a complete cycle of movement starting at 90° of hip flexion in the sitting position, continuing knee flexion to 135°, and then returning to 90° of knee flexion. The movement can be approximated as a single plane movement in the sagittal plane; therefore, the angle between the thigh and shin during knee flexion was calculated using three marker points: the lateral ankle, lateral tibial condyle, and upper 2/3 of the line connecting the lateral tibial condyle to the greater trochanter. Based on this angle, MATLAB was used to divide flexion and extension movements. The position where the angle is 2% above the minimum angle was used as the end point of the flexion action and the starting point of the extension action, and the position where the angle is 98% above the maximum angle was used as the end point of the extension action and start point of the flexion action, thus defining the flexion and extension processes in a single motion cycle.

### Kinesio taping techniques

**Fig. 2:**
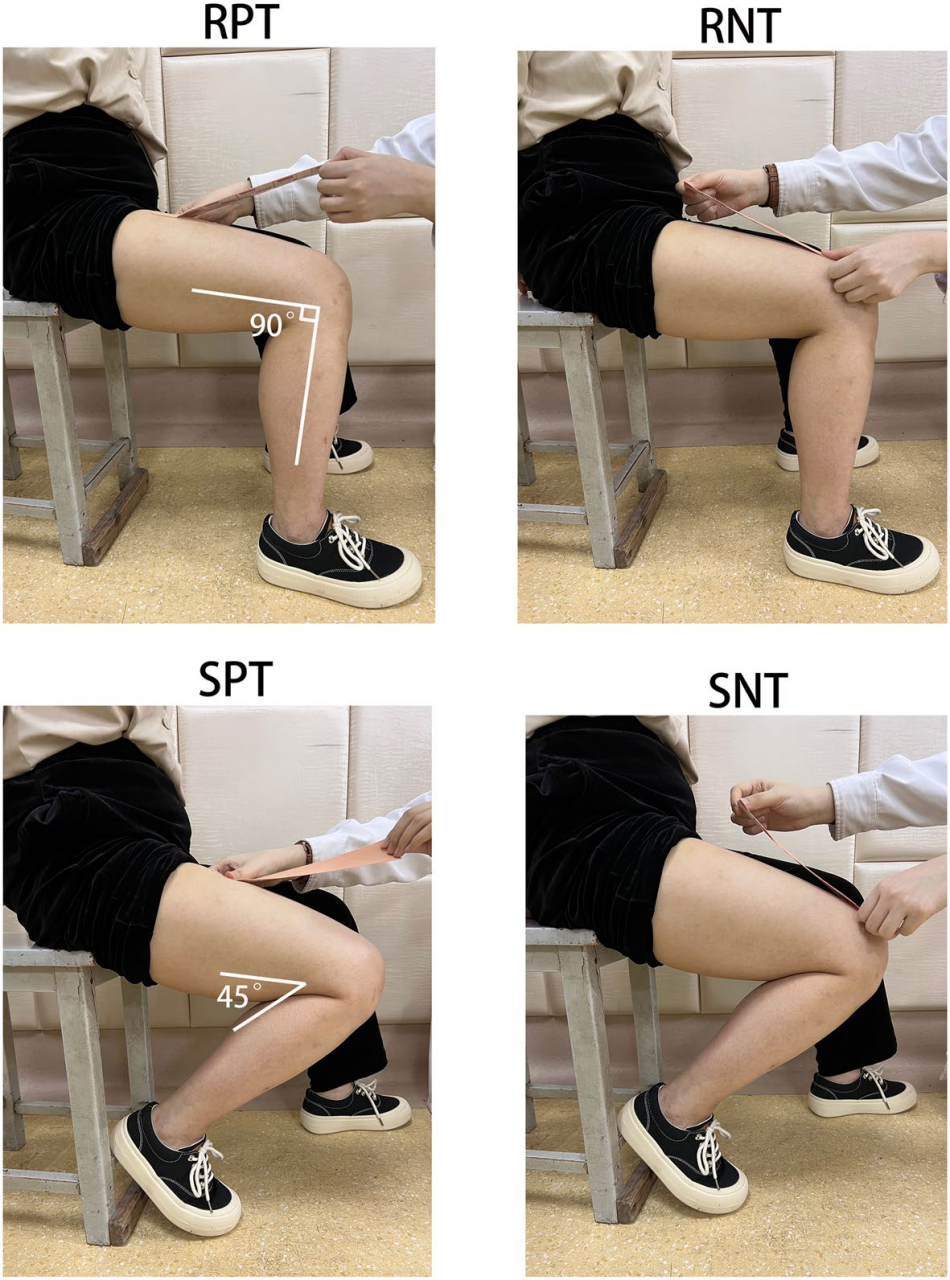
Taping methods. RPT, resting positive taping; RNT, resting negative taping; SPT, stretching positive taping; SNT, stretching negative taping

After fixation of the reflective marker, the subjects were placed in a sitting position. The motion data were first collected without KT from the starting position (90° of hip and knee flexion) to 135° of knee flexion and then back to the starting position, in a 2-s exercise cycle. This motion was repeated five times to the rhythm of a metronome. Subsequently, a 25-cm section of I-tape was applied to the anterior thigh skin surface under natural tension. The reflective markers were applied 1 cm from each edge of the patch, as shown in Fig. 1a-b. The taping methods included resting quadriceps taping and stretching of the quadriceps taping. Resting quadriceps taping consisted of taping at 90° of knee flexion, including RPT (resting positive taping) and RNT (resting negative taping). Stretching quadriceps taping consisted of taping at 135° of knee flexion, including SPT (stretching positive taping) and SNT (stretching negative taping). The anchor point of the positive taping was located at the proximal trunk, and the anchor point of the negative taping was located at the distal trunk. As shown in Fig. 2. Measurements of anterolateral thigh skin deformation were performed for subjects experiencing NT, RPT, RNT, SPT, and SNT. All subjects were required to complete five complete motion cycles for data acquisition. The exercise procedure and data collection were performed as described previously.

### Statistical analysis

The characteristics of subjects were described using mean ± SD values for continuous variables, and numbers for categorical variables. Concordance of variables with a normal distribution was tested using Shapiro–Wilk. Data were expressed as mean ± standard deviation (‾x ± s). The dynamic change of the angle between the upper and lower legs when the knee is flexed or extended is represented by a line graph. The inter-group comparison of the distance and total length of skin measurement points under different taping methods used a mixed effects model. The fixed effect in the model is the test time (five time points T1-T5 are selected at equal intervals according to the degree of movement completion, the first frame of a motion cycle obtained by dynamic capture is T1, and the last frame is T5), random effects are different taping methods (NT, RPT, RNT, SPT, SNT). SAS 9.4 (SAS Institute, Cary, NC, USA) was used for data analysis, and Matlab was used for graph drawing. The significance level is 0.05.

## Results

### Changes in skin length of the subject’s right anterior thigh

The cohort included ten subjects ( 4 men: age 25.25 ± 0.96 years, height 168.50 ± 3.11 cm, weight 69.50 ± 10.21 kg. 6 women: age 27.33 ± 3.56 years, height 164.50 ± 3.27 cm, weight 54.67 ± 4.32 kg)(Table 1).

**Table 1:** Physical characteristics of the subjects

	**Males (*n* = 4)**	**Females (*n* = 6)**
Height (cm)	168.50 ± 3.11	164.50 ± 3.27
Weight (kg)	69.50 ± 10.21	54.67 ± 4.32
Age (years)	25.25 ± 0.96	27.33 ± 3.56

**Fig. 3:**
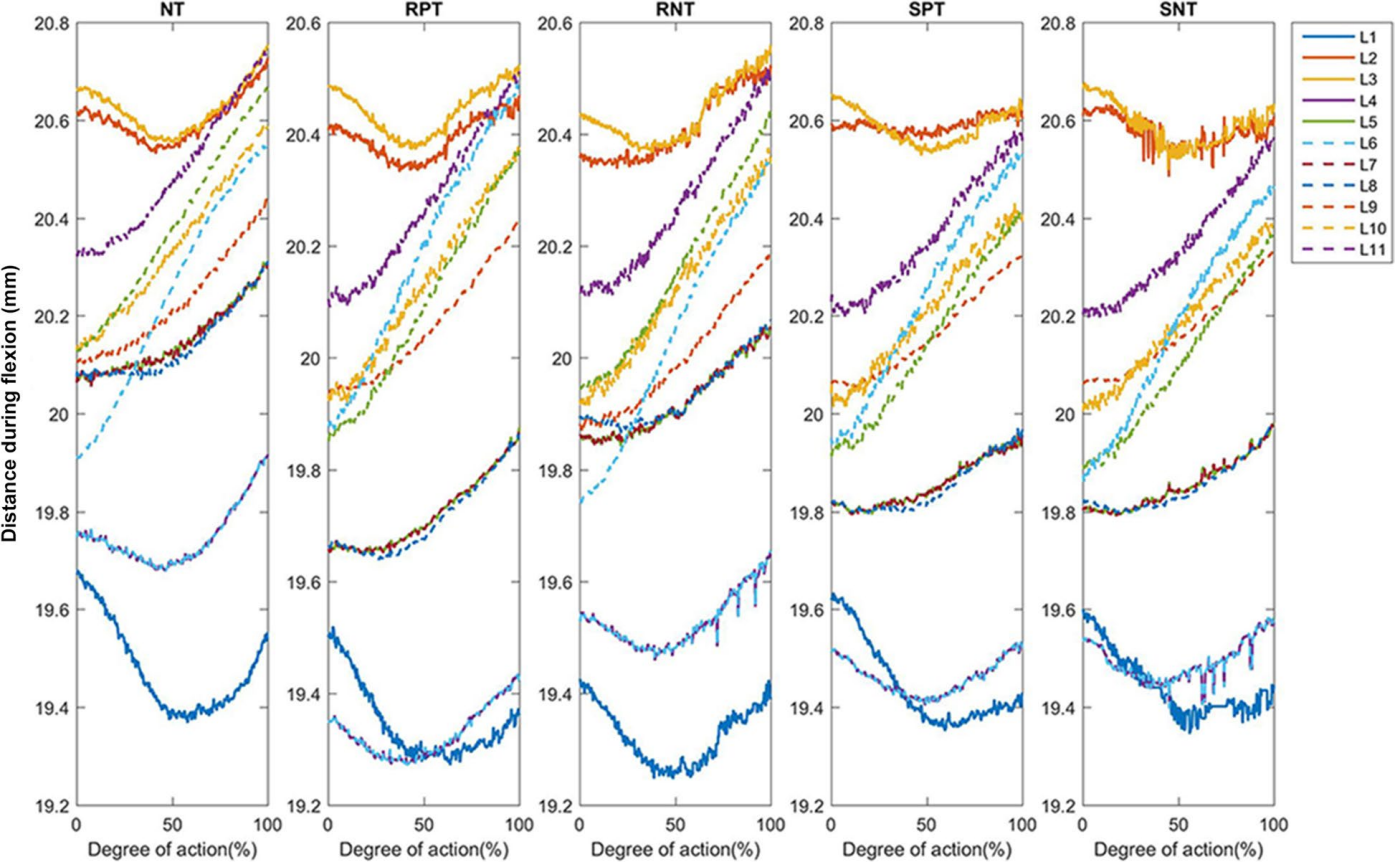
Changes in the distance between adjacent points during flexion. NT: no Kinesio taping; RPT, resting positive taping; RNT, resting negative taping; SPT, stretching positive taping; SNT, stretching negative taping. L1–L11 represent the length of the distance between two adjacent reflective markers (R1–R12)

**Fig. 4:**
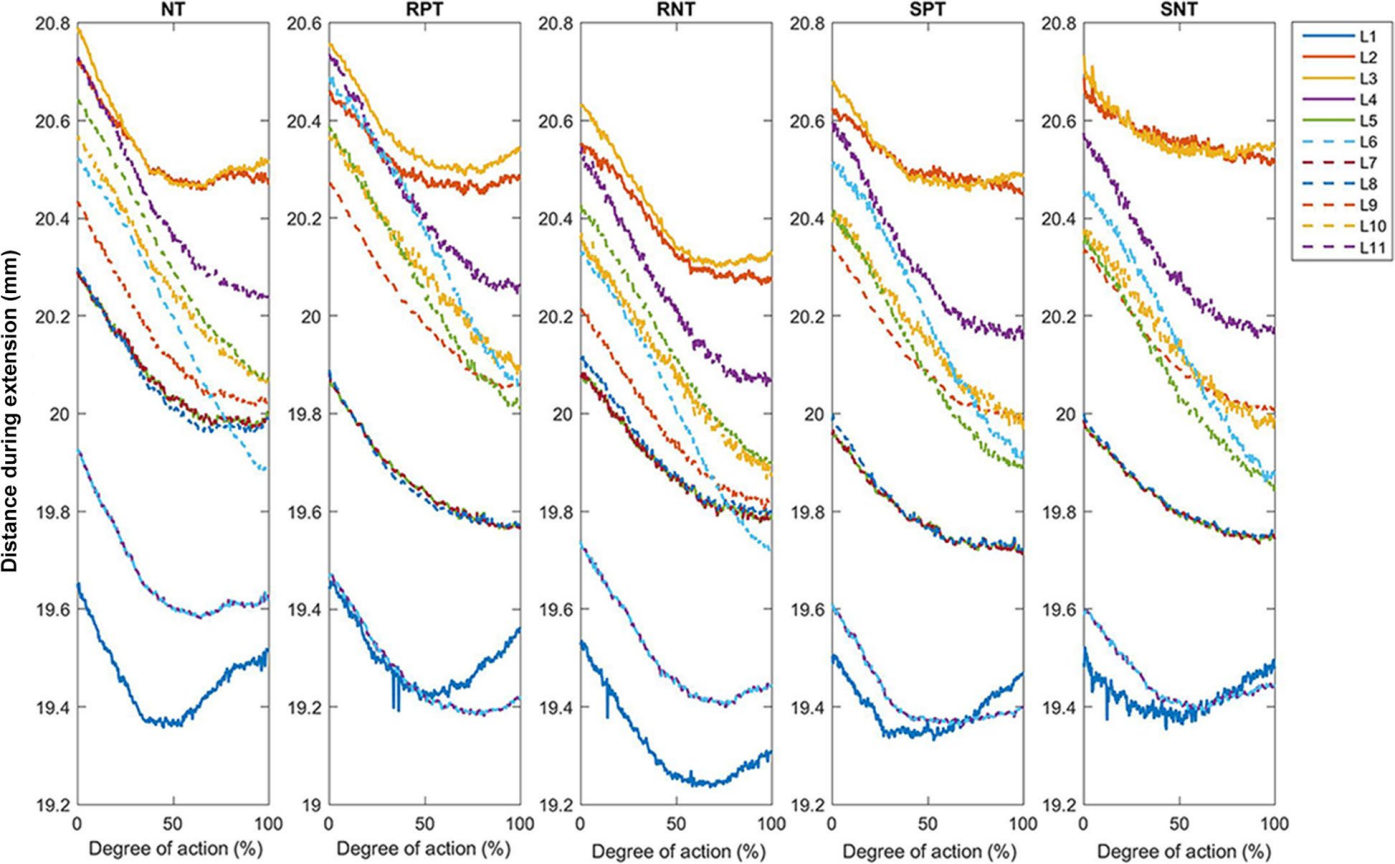
Changes in the distance between adjacent points during extension. NT: no Kinesio taping; RPT, resting positive taping; RNT, resting negative taping; SPT, stretching positive taping; SNT, stretching negative taping. L1–L11 represent the length of the distance between two adjacent reflective markers (R1–R12)

The results from Figs. 3 and 4 suggest that the 3-dimensional coordinated capture system accurately located the positions of the reflective markers. The lengths of the L1–L11 segments showed a similar tendency with increasing range of motion in flexion (Fig. 3) and extension (Fig. 4) under NT, RPT, RNT, SPT, and SNT conditions. However, slight differences were observed in the degree of skin deformation under different conditions.

To further analyze the effects of Kinesio taping on skin deformation, five time points (T1–T5) were selected according to the degree of movement completion during right knee flexion and extension. Based on the mixed-effects models, the changes in L0 (total length), L1 (distal to the knee), L6 (intermediate segment), and L11 (proximal to the knee segment) were compared. The obtained results revealed that except for the length of L1 during flexion, where the difference between groups was not significant (*P* > 0.05), the differences in skin deformation were significant between all groups (*P* < 0.05), suggesting that during the dynamic process of flexion (Table 2) and extension (Table 3), skin deformation was affected by Kinesio taping and taping methods.

**Table 2:** Length changes in L0, L1, L6, and L11 at 5 different time points during right knee flexion in 10 subjects subjected to NT, RPT, RNT, SPT, and SNT

^A^No	^B^Time	^C^NT	^D^RPT	^E^RNT	^F^SPT	^G^SNT	^H^*P*
**L0**	**T1**	218.46 ± 6.40	216.06 ± 6.40	216.14 ± 6.38	217.36 ± 6.26	217.11 ± 6.04	< 0.0001
	**T2**	219.49 ± 6.05	217.05 ± 6.25	216.92 ± 6.06	218.20 ± 5.93	217.95 ± 5.44	
	**T3**	220.96 ± 5.58	218.70 ± 5.85	218.54 ± 5.36	219.60 ± 5.16	219.48 ± 4.71	
	**T4**	222.55 ± 5.37	220.36 ± 5.43	220.27 ± 4.85	221.26 ± 4.78	220.96 ± 4.47	
	**T5**	224.02 ± 5.43	221.70 ± 5.23	221.63 ± 4.71	222.39 ± 4.80	222.23 ± 4.54	
**L1**	**T1**	19.67 ± 2.15	19.51 ± 2.17	19.42 ± 2.18	19.62 ± 2.07	19.60 ± 2.04	0.0733
	**T2**	19.53 ± 2.05	19.37 ± 2.09	19.32 ± 2.10	19.51 ± 2.02	19.40 ± 1.95	
	**T3**	19.40 ± 2.01	19.29 ± 2.02	19.26 ± 2.03	19.38 ± 1.93	19.37 ± 1.84	
	**T4**	19.41 ± 1.99	19.30 ± 1.96	19.34 ± 1.99	19.38 ± 1.88	19.38 ± 1.80	
	**T5**	19.54 ± 1.93	19.36 ± 1.92	19.39 ± 1.95	19.43 ± 1.84	19.45 ± 1.80	
**L6**	**T1**	20.08 ± 1.64	19.66 ± 1.67	19.89 ± 1.78	19.82 ± 1.71	19.82 ± 1.63	< 0.0001
	**T2**	20.08 ± 1.63	19.65 ± 1.63	19.88 ± 1.75	19.81 ± 1.69	19.80 ± 1.59	
	**T3**	20.10 ± 1.60	19.68 ± 1.58	19.90 ± 1.67	19.82 ± 1.60	19.83 ± 1.48	
	**T4**	20.19 ± 1.60	19.76 ± 1.55	19.97 ± 1.63	19.90 ± 1.56	19.89 ± 1.45	
	**T5**	20.31 ± 1.62	19.87 ± 1.52	20.07 ± 1.62	19.97 ± 1.55	19.98 ± 1.47	
**L11**	**T1**	19.91 ± 0.78	19.88 ± 0.77	19.75 ± 0.70	19.95 ± 0.70	19.87 ± 0.73	< 0.0001
	**T2**	20.05 ± 0.80	20.02 ± 0.77	19.86 ± 0.69	20.07 ± 0.68	20.00 ± 0.71	
	**T3**	20.26 ± 0.81	20.21 ± 0.79	20.05 ± 0.70	20.25 ± 0.71	20.20 ± 0.74	
	**T4**	20.44 ± 0.83	20.35 ± 0.80	20.22 ± 0.73	20.41 ± 0.73	20.34 ± 0.75	
	**T5**	20.55 ± 0.84	20.48 ± 0.84	20.35 ± 0.76	20.53 ± 0.77	20.47 ± 0.77	

^A^No: L0, distance between points R1 and R12; L1, distance between points R1 and R2; L6, distance between points R6 and R7; L11, distance between points R11 and R12

^B^Time: five time points (T1, starting; T5, finishing) in the process of completing the motion

^C^NT: no Kinesio taping; ^D^RPT, resting positive taping; ^E^RNT, resting negative taping; ^F^SPT, stretching positive taping; ^G^SNT, stretching negative taping

^H^*P*, *P*-values were calculated by the mixed-effects models

**Table 3:** Length changes in L0, L1, L6, and L11 at 5 different time points during right knee extension in 10 subjects subjected to NT, RPT, RNT, SPT, and SNT

^A^No	^B^Time	^C^NT	^D^RPT	^E^RNT	^F^SPT	^G^SNT	^H^*P*
**L0**	**T1**	224.05 ± 5.53	221.92 ± 5.51	221.96 ± 5.13	222.64 ± 4.97	222.36 ± 4.86	< 0.0001
	**T2**	222.07 ± 5.38	220.04 ± 5.63	220.19 ± 5.53	220.85 ± 4.98	220.66 ± 4.95	
	**T3**	219.95 ± 5.53	217.94 ± 5.82	218.01 ± 5.88	218.85 ± 5.39	218.75 ± 5.26	
	**T4**	218.35 ± 5.91	216.19 ± 5.92	216.24 ± 5.93	217.30 ± 5.73	217.36 ± 5.64	
	**T5**	217.43 ± 6.17	215.32 ± 6.08	215.38 ± 6.03	216.57 ± 5.98	216.56 ± 5.86	
**L1**	**T1**	19.65 ± 1.92	19.45 ± 1.96	19.53 ± 1.96	19.50 ± 1.86	19.49 ± 1.82	0.0486
	**T2**	19.45 ± 1.96	19.30 ± 1.98	19.38 ± 2.00	19.37 ± 1.88	19.41 ± 1.87	
	**T3**	19.37 ± 2.03	19.23 ± 2.00	19.25 ± 2.02	19.36 ± 1.95	19.39 ± 1.94	
	**T4**	19.45 ± 2.12	19.26 ± 2.09	19.24 ± 2.06	19.38 ± 2.02	19.43 ± 2.00	
	**T5**	19.52 ± 2.13	19.36 ± 2.15	19.31 ± 2.14	19.47 ± 2.03	19.48 ± 2.03	
**L6**	**T1**	20.30 ± 1.57	19.89 ± 1.53	20.11 ± 1.65	19.99 ± 1.58	20.00 ± 1.50	< 0.0001
	**T2**	20.14 ± 1.57	19.71 ± 1.53	19.99 ± 1.68	19.85 ± 1.56	19.88 ± 1.49	
	**T3**	20.01 ± 1.59	19.63 ± 1.53	19.87 ± 1.67	19.77 ± 1.58	19.80 ± 1.50	
	**T4**	19.98 ± 1.63	19.58 ± 1.58	19.81 ± 1.68	19.73 ± 1.63	19.76 ± 1.54	
	**T5**	19.99 ± 1.62	19.57 ± 1.58	19.81 ± 1.72	19.72 ± 1.63	19.76 ± 1.56	
**L11**	**T1**	20.52 ± 0.84	20.48 ± 0.86	20.33 ± 0.78	20.51 ± 0.79	20.45 ± 0.79	0.0001
	**T2**	20.38 ± 0.84	20.35 ± 0.82	20.19 ± 0.75	20.38 ± 0.75	20.32 ± 0.77	
	**T3**	20.20 ± 0.83	20.17 ± 0.79	20.00 ± 0.74	20.19 ± 0.72	20.13 ± 0.75	
	**T4**	20.00 ± 0.80	19.98 ± 0.79	19.83 ± 0.72	20.01 ± 0.71	19.99 ± 0.75	
	**T5**	19.87 ± 0.78	19.85 ± 0.77	19.72 ± 0.72	19.91 ± 0.71	19.87 ± 0.74	

^A^No: L0, distance between points R1 and R12; L1, distance between points R1 and R2; L6, distance between points R6 and R7; L11, distance between points R11 and R12

^B^Time: five time points (T1, starting; T5, finishing) in the process of completing the motion

^C^NT: no Kinesio taping; ^D^RPT, resting positive taping; ^E^RNT, resting negative taping; ^F^SPT, stretching positive taping; ^G^SNT, stretching negative taping

^H^*P*, *P*-values were calculated by the mixed-effects models

### Changes in L0, L1, L6, and L11 of subjects’ right anterior thigh

**Fig. 5:**
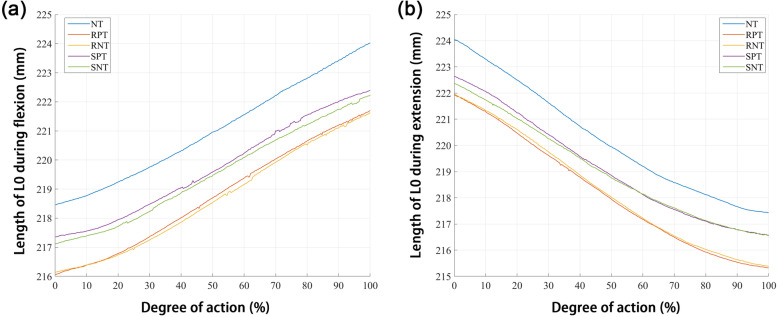
Change in total length (L0) during (**a**) flexion and (**b**) extension. NT: no Kinesio taping; RPT, resting positive taping; RNT, resting negative taping; SPT, stretching positive taping; SNT, stretching negative taping

**Fig. 6:**
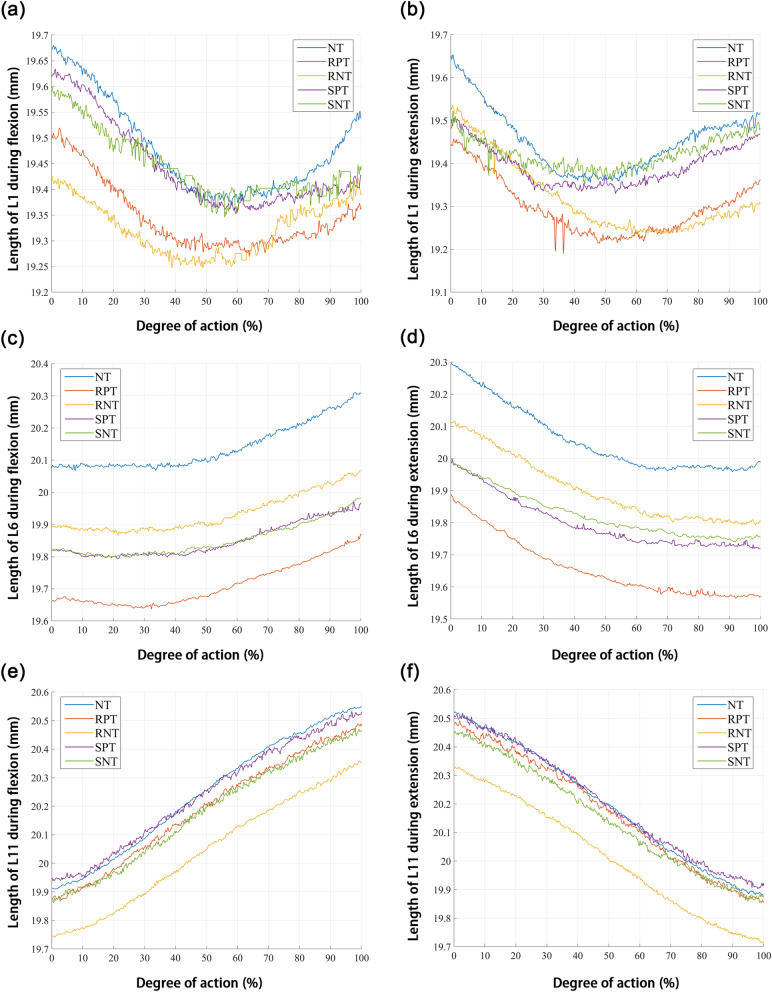
Change in length of L1, L6 and L11 during flexion and extension. Length of L1 during (**a**) flexion and (**b**) extension. Length of L6 during (**c**) flexion and (**d**) extension. Length of L11 during (**e**) flexion and (**f**) extension. L1 represents the gap between R1 and R2, L6 the gap between R6 and R7, and L11 the gap between R11 and R12. NT: no Kinesio taping; RPT, resting positive taping; RNT, resting negative taping; SPT, stretching positive taping; SNT, stretching negative taping

In terms of L0 length during right knee flexion and extension, L0 length under NT conditions was longer than that under other conditions (Fig. 5a-b). Moreover, the taping in the stretching position increased the length of L0 compared to that when taping in the resting position (Fig. 5a-b). During right knee flexion and extension, L1 (Fig. 6a-b), L6 (Fig. 6c-d), and L11 (Fig. 6e-f) lengths were longer in the NT group than in the other groups. Compared to taping in the resting position, taping in the stretching position increased the length of L1 (Fig. 6a-b) and L11 (Fig. 6e-f).

## Discussion

In this study, we demonstrated that skin deformation was affected differently by kinesio taping and taping methods. The total length (L0) and segment lengths (L1, L6, and L11) in the NT group were longer than those in the RPT, RNT, SPT, and SNT groups, regardless of right knee flexion or extension, indicating that Kinesio application resulted in shorter skin distances. In addition, during right knee flexion and extension, L0, L1, and L11 lengths were longer in the stretching position than in the rest position. However, there seemed to be no similar patterns in the length changes obtained following negative or positive taping. This suggests that limb position may affect the effects of Kinesio tape on dynamic skin deformation, while the change in taping direction does not seem to have a significant effect.

According to the traditional theory of Kinesio taping, stress and tangential forces are two different directions of action of the patch, i.e., downward or horizontal forces [21, 22]. When combined with the taping position, the patch can also produce an upward grip on the skin [23]. The type of force that plays a role depends on the tension of the taping to some extent [24]. Usually, the greater the local tissue stress induced by the taping, the greater the vertical effect exerted on the target tissue, and the greater the pressure and support of the target tissue, such as mechanical correction of ligament taping, mainly through the downward force generated by the large stress [25]. When the vertical stress of the patch is small, the horizontal tangential force is easily generated to drive the fascia and subcutaneous tissues, the skin is easily grabbed and produces folds, and the interstitial fluid and circulation metabolism are accelerated [26]. Commonly used lymphatic taping and neuromuscular taping play roles through stress-induced horizontal and upward grasping forces [27]. Natural tension I-tape was used in our study. The results show that this form of taping shortens the skin distance between the two ends of the taping which is consistent with the theory that when the vertical stress of the tape is small, taping can cause the skin to be pulled up and produce folds. In addition, our study showed that taping under different limb positions had a greater impact on the change of dynamic skin deformation. While under the same positioning, different taping directions did not seem to have a significant effect on skin deformation. This shows that kinesio taping does have an impact on the biomechanical changes of the skin, but it does not support the concept of taping direction in the traditional taping theory. In the 1990s, Europe and the United States and Taiwan, China did a lot of research on the concepts of "anchor", "tail" and taping direction [20]. Some researchers believe that when the tape is applied from the starting point of the muscle to the end point (the retraction direction of the tape is the same as that of the muscle contraction), it can promote muscle contraction. On the contrary, when the tape is applied from the end point of the muscle to the starting point (the direction of retraction of the tape is opposite to the direction of muscle contraction), it will have a relaxing effect, which can inhibit the excessive tension caused by excessive use of the muscle, and shows a protective effect [28, 29]. However, in recent years, several studies have shown that the taping direction has no significant effect on the treatment outcome. A study by Choi et al. [30] to determine the effect of taping orientation on quadriceps muscle strength under fatigue showed that regardless of taping orientation, muscle strength under fatigued conditions could be improved. Another study has shown that taping in different directions does not adjust the reflex excitability of muscles, nor does it have different effects on muscle strength and joint mobility [31]. A study done in China showed that there was no obvious correlation between the taping direction and the change of the subcutaneous space, which also confirmed this point of view [32]. This is also in line with some monographs emphasizing placement, tension, and weakening the direction of taping.

We found that during right knee flexion, the deformation of L1 segment skin was not significantly different between the 10 subjects, which may be related to the fact that the L1 segment was the observed section furthest from the knee. In addition, it has been found that skin stretching changes in each region of the leg during cycling, mainly in the outer thigh region and especially in the knee region [33]. The target movements selected for this study were knee flexion and extension. The skin deformation of the L1 segment was small because of far distance from the active part of the joint; therefore, it is possible that the effect of Kinesio taping on its deformation was not significant. Additionally, the skin deformation of the L6 segment seemed to be unrelated to patch placement. The causative factor may be that L6 is the middle of the active area. We conjecture that the effect of limb position during taping may be mainly manifested at the two ends of the taping area, which needs to be verified in further studies. Moreover, the small number of subjects in this study is a possible reason for the above situation.

In this study, the effects of Kinesio taping on skin deformation are actually the result of a change in the stress–strain of the skin, suggesting that Kinesio taping may play a pre-stressing role in the dynamic deformation of the skin. The skin, consisting of the epidermis and dermis, can be regarded as being composed of an elastic gel and collagen fibers [34]. At low stress, collagen fibers are in a relaxed state [35]. As the stress increases, some fibers start to become taut and stressed, and the elastic modulus starts to increase [36]. At high stress, all fibers are stressed and exhibit a high stiffness and elastic modulus [36, 37]. Not all collagen fibers are stressed at the same time during the stretching process, and the fiber distribution has obvious directionality; therefore, the overall skin stress shows non-linearity and anisotropy [37]. In our study, Kinesio taping was introduced; some collagen fibers in the dermis contract under the action of ligation, stretching from a flexed and relaxed state to an extended state, and start to bear certain tensions. Hence, the effect of Kinesio taping is equivalent to applying pre-stress to the fibers, stressing the collagen fibers in advance and releasing some of their strength reserves. Taping is like applying an extra layer of protection to the skin, which explains that compared with the non-applied group, all types of taping shorten the overall length and the spacing of each section. It has been pointed out that the shape of the skin stress–strain curve is not the same depending on whether the experiment is performed in vivo or in vitro, and that this difference is related to the initial stress in the skin. Similarly, the positioning before taping in this study is equivalent to changing the initial stress of the skin, and this initial stress interacts with the "pre-stress" generated by kinesio taping, which shows an added-on effect of skin deformation during exercise.

Many limitations are found in this study, including the state of the instrumentation and number and age of the subjects. Further expanding the sample size and obtaining a reasonable range of data is needed in future studys. A better use of the motion capture system to further investigate the effect of Kinesio taping on skin deformation to build up on the mechanisms of taping is also a must.

## Conclusions

The Kinesio tape does have an impact on the biomechanical changes of the skin, thus resulting in skin deformation. The use of natural tension I-type taping can shorten the total length of the skin between the two ends of the taping and the distance between each section. The limb position during taping may be a factor interfering the taping effect, and the change of taping direction does not seem to have a significant effect on the skin deformation during exercise. The mechanism of why does the Kinesio tape work may be that it acts as a "pre-stress" in the biomechanics of the skin.

## Data Availability

All data generated or analysed during this study are included in this published article and its supplementary information files.

## Abbreviation

KT: Kinesio taping
